# Presumptive paroxysmal dyskinesia in 41 dogs: is the diet change enough?

**DOI:** 10.1093/jvimsj/aalag238

**Published:** 2026-10-09

**Authors:** Henry Mendo Reyes, Greta Galli, Evelina Burbaitè, Aurora Cocchetto, Erica Fiorentino, Marika Menchetti

**Affiliations:** Neurology Division, San Marco Veterinary Clinic and Laboratory, Veggiano, Italy; Neurology Division, San Marco Veterinary Clinic and Laboratory, Veggiano, Italy; Neurology Division, San Marco Veterinary Clinic and Laboratory, Veggiano, Italy; Dr. L. Kriauceliunas Small Animal Clinic, Lithuanian University of Health Sciences, Kaunas, Lithuania; Neurology Division, San Marco Veterinary Clinic and Laboratory, Veggiano, Italy; Neurology Division, San Marco Veterinary Clinic and Laboratory, Veggiano, Italy; Neurology Division, San Marco Veterinary Clinic and Laboratory, Veggiano, Italy

**Keywords:** celiac disease, dystonia, gluten-free diet, gut–brain axis, nonceliac gluten sensitivity

## Abstract

**Background:**

Paroxysmal dyskinesia (PD) in dogs comprises episodic, involuntary, nonepileptic movement disorders. Recent evidence suggests that PD may be associated with gluten sensitivity, paralleling human gluten-related neurologic syndromes.

**Hypothesis/Objectives:**

Characterize clinical features, gastrointestinal comorbidities, gluten-related serology, and response to a gluten-free diet (GFD) in dogs with presumptive gluten-sensitive PD. We hypothesized that dietary modification would decrease episode frequency, particularly in dogs with gastrointestinal signs, seropositivity for anti-gliadin and anti-transglutaminase-2 immunoglobulin A antibodies or both.

**Animals:**

Forty-one client-owned dogs with presumptive PD.

**Methods:**

Retrospective review of dogs diagnosed with presumptive PD at a single institution between 2019 and 2023. Inclusion criteria included > 1 episode, video-documented episodes, normal interictal neurologic examination, serum gluten-related antibody titers, and ≥ 6 months of follow-up after transition to GFD.

**Results:**

Gastrointestinal signs were reported in 33/41 (80.5%) dogs; 21/41 (51.2%) had at least 1 positive antibody titer. After dietary change, 24/41 (58.5%) dogs achieved complete resolution, 15/41 (36.6%) showed partial response, and 2/41 (4.9%) remained unchanged, regardless of antibody status. All owners reported gastrointestinal improvement. Nine dogs relapsed after initial response: 6 after gluten ingestion, 1 associated with stress, and 2 despite strict GFD. Response to GFD was not significantly associated with gastrointestinal signs or antibody status.

**Conclusions and clinical importance:**

Although dietary response was not significantly associated with antibody status or gastrointestinal signs, GFD led to substantial clinical improvement in most dogs with PD across multiple breeds, supporting a potential link between gluten sensitivity and PD, and a possible role of the gut–brain axis in disease pathogenesis.

## Introduction

Paroxysmal dyskinesia (PD) in dogs is a heterogeneous group of movement disorders characterized by recurrent episodes of abnormal, hyperkinetic, involuntary movements, typically without autonomic signs or loss of consciousness. The underlying pathophysiologic mechanism is currently unknown, but dysfunction of the basal nuclei, cerebellum, or both has been considered.^1^ Clinical diagnosis is based on a detailed description or visualization of the episodes or both, a normal interictal neurologic examination, and exclusion of other paroxysmal events, particularly epileptic seizures. Additional diagnostic tests, such as brain magnetic resonance imaging (MRI) and cerebrospinal fluid (CSF) analysis, are commonly performed to exclude structural diseases. However, these investigations are not systematically undertaken, especially when the neurologic examination is normal and video recordings are compatible with PD. Electroencephalography (EEG) may support the diagnosis of PD, especially in challenging cases, but is not routinely performed.^1–3^

In human medicine, PD is categorized according to the predominant movement pattern, including ballism, dystonia, chorea, or athetosis.^4^^,^^5^ In veterinary medicine, because of anatomical differences between species, the International Veterinary Canine Dyskinesia Task Force recommends using only the terms dyskinesia and dystonia.^1^ Paroxysmal dyskinesia in dogs can be classified etiologically as primary, when a genetic basis has been confirmed (eg, Cavalier King Charles Spaniel, Soft-Coated Wheaten Terrier), or suspected (eg, Scottish Terrier, Labrador Retriever, and Jack Russell Terrier), or as secondary, when associated with drug administration (eg, propofol), structural intracranial disease, or gluten sensitivity.^1^^,^^6–9^

Several therapeutic approaches have been attempted in dogs, including antiseizure medication (ASM), acetazolamide, fluoxetine, and dietary modification, with variable success.^2^^,^^10^^,^^11^ An exception is the Border Terrier, in which gluten-sensitive PD has been recognized, associated with increased serum anti-gliadin immunoglobulin G (AG) titers, anti-transglutaminase-2 immunoglobulin A (ATG2-A) antibody titers, or both, and a favorable response to a gluten-free diet (GFD).^12–14^

In humans, gluten-related disorders such as nonceliac gluten sensitivity (NCGS) and celiac disease (CD) have been associated with various movement disorders, and the role of the gut–brain axis (GBA) has gained increasing attention in recent decades.^5^^,^^15–17^ Clinical improvement after dietary modification has been reported in other breeds of dogs, but current evidence remains limited to establish a definitive association.^18^^,^^19^

Our aim was to characterize the clinical features, gastrointestinal comorbidities, gluten-related serology, and response to GFD in dogs with presumptive gluten-sensitive PD. We hypothesized that dietary modification would decrease episode frequency, particularly in dogs with gastrointestinal signs, seropositivity for AG and ATG2-A antibodies or both.

## Materials and methods

A retrospective, single-center cohort study was conducted between January 2019 and September 2023.

Dogs were included if they met the following inclusion criteria:

a) complete medical records including clinical history, signalment, diet information, concurrent medical conditions, and physical and neurologic examination findings;
b) a presumptive clinical diagnosis of PD based on > 1 episode, video recordings reviewed by a European College of Veterinary Neurology (ECVN) diplomate, normal interictal neurologic examination performed by an ECVN diplomate or a supervised neurology resident, and unremarkable blood test results;
c) serum AG and ATG2-A antibody titers measured at diagnosis;
d) implementation of a strict GFD; and,
e) ≥6 months of follow-up after dietary modification.

Abdominal ultrasonography, brain MRI, computed tomography (CT), and CSF analysis were reviewed when available.

Episode frequency, potential triggers, and gastrointestinal and autonomic signs were recorded. Episode frequency was categorized as daily, weekly, monthly, or yearly.

Follow-up was categorized as short-term (1-3 months) or long-term (≥6 months). All owners were interviewed by telephone or in person at the end of the study period. Response to diet was classified as complete resolution or partial response, the latter defined as a decrease in episode frequency. Relapse was defined as recurrence or worsening of episodes after initial improvement after GFD implementation.

Serum AG and ATG2-A antibody titers were measured using standardized ELISA assays at external laboratories. Until December 2020, samples were analyzed at Veterinary Pathology Group (Hitchin, UK), which classified results as positive if AG > 0.25 and ATG2-A > 0.41. After December 2020, samples were submitted to Laboklin (Bad Kissingen, Germany), where AG and ATG2-A > 0.8 were considered positive, 0.6-0.8 equivocal, and < 0.6 negative. Seropositivity was defined as positivity for at least 1 antibody, whereas seronegativity was defined as being negative for both antibodies. Statistical analyses were repeated considering questionable results as either positive or negative.

Data analysis was performed using PAST version 5.2 (Paleontological Statistics, Oslo, Norway). Continuous variables such as age and body weight were summarized using means. Associations between categorical variables, including antibody status, presence of gastrointestinal signs, episode frequency, diet type (commercial or home-prepared), and response to GFD were assessed using the Chi-squared test, or Fisher’s exact test when ≥ 1 expected cell count in the contingency table was ≤ 5. For larger contingency tables with expected cell counts < 5, Monte Carlo simulation of *P*-value was used. *P*-values < .05 were considered statistically significant. Cramer’s V was calculated to assess the strength of association.

## Results

### Study population

A total of 68 dogs were diagnosed with PD between January 2019 and September 2023; of these, 41 met the inclusion criteria.

The mean age at onset of the first episode was 4.1 years (range, 0.5-9 years) and mean age at diagnosis was 5.6 years (range, 0.5-10.8 years). Mean body weight was 9.4 kg (range, 1.0-35.4 kg).

Males were more commonly represented (26/41, 63.4%; 18 intact, 8 neutered) than females (15/41, 36.6%, 5 intact, 10 neutered).

The most commonly represented breeds were crossbreeds (9/41, 22%), Pomeranians (7/41, 17.1%), Maltese (7/41, 17.1%), and Poodles (7/41, 17.1%). Other breeds included 4 Labrador Retrievers, 3 French Bulldogs, 2 Jack Russell Terriers, and 1 each of Chihuahua and Cavalier King Charles Spaniel.

### Prior comorbidities and treatments

Concurrent medical disorders were diagnosed in 23/41 dogs (56.1%). Reported conditions included chronic enteropathy (3), chronic gastroenteropathy (8), dermatitis with gastrointestinal signs (2), dermatitis alone (4), intestinal malabsorption (1), gastritis (2), gastrointestinal parasites (1), cardiomyopathy (1), and hypothyroidism (1).

Medical treatment before referral had been initiated in 10/41 dogs. Antiseizure medications were used in 4/10 dogs and included diazepam (1), imepitoin (1), imepitoin followed by phenobarbital (1), and phenobarbital (1), with no reported clinical improvement in any of the cases. Other medications included ursodeoxycholic acid (1 dog with chronic enteropathy), omeprazole (1 dog with chronic gastroenteropathy), sucralfate (in 1 dog with gastritis), oclacitinib (1 dog with dermatitis alone), ceftazidime with corticosteroids (1 dog with dermatitis alone), and levothyroxine (1 dog with hypothyroidism).

### Gastrointestinal clinical signs and diet information

Gastrointestinal signs were reported in 33/41 dogs (80.5%; Table 1).

**Table 1:** Clinical characteristics, gastrointestinal signs, and gluten-related antibody titers in 41 dogs with PD.

Variable	*n* (%)
**Episode frequency (*n* = 41)**	
**Daily**	10 (24.4)
**Weekly**	5 (12.2)
**Monthly**	18 (43.9)
**Yearly**	8 (19.5)
**Gastrointestinal signs (*n* = 33)**	
**Vomiting**	22 (66.7)
**Diarrhea**	16 (48.5)
**Borborygmi**	11 (33.3)
**Soft feces**	6 (18.2)
**Nausea**	3 (9.1)
**Antibody titers** ^**a**^ **(*n* = 41)**	
**Both antibodies positive**	5 (12.2)
**One antibody positive (AG or ATG2-A)**	16 (39)
**Both antibodies negative**	20 (48.8)

Abbreviations: AG = anti-gliadin immunoglobulin G; ATG2-A = anti-transglutaminase-2 immunoglobulin A; PD = paroxysmal dyskinesia.

a Questionable results were considered as negative.

At presentation, 2/41 dogs were already being fed a strict commercial GFD. Among the remaining 39 dogs, diet types included various brands of commercial diets containing gluten (28), prescription diets containing gluten (6), hypoallergenic diets that were gluten-free (3), and home-prepared diets (2; 1, gluten-free). Of these 39 dogs, 35 were also receiving gluten-containing treats, foods for humans or both, including all 3 dogs fed hypoallergenic diets and the dog receiving a gluten-free home-prepared diet.

### Dyskinesia phenotype

A detailed description of episodes for each dog is provided in Table S1.

The mean episode duration was 19 min (range, 0.5-240 min). Episode frequency was variable: daily in 10 dogs, weekly in 5, monthly in 18, and yearly in 8 (see Table 1).

Regarding specific movement features, which were not mutually exclusive, 17/41 dogs (41.5%) showed dystonic movements of all four limbs, 11/41 (26.8%) had whole body involvement, 10/41 (24.4%) predominantly affected the pelvic limbs, 6/41 (14.6%) displayed alternating movements of a single leg, and 6/41 (14.6%) had dystonic head tremors.

All owners reported preserved awareness during episodes; all dogs responded to call or touch, although only 12 attempted to reach the owner. All cases exhibited difficulty walking during episodes. Behavioral changes were reported in 27/41 dogs (65.9%): 15 appeared anxious, 2 painful, and 10 both.

Autonomic signs were observed in 8/41 dogs (19.5%). Salivation was the most common sign (7/8), characterized in all affected dogs by minimal droplet loss of saliva, followed by urination (4/8), described by owners as a small amount of urine leakage. In 5/7 dogs with salivation, gastrointestinal signs were also reported.

One dog was described as mildly apathetic after episodes.

Potential triggers were reported in 7/41 dogs (17.1%), including stress or excitement (eg, doorbell ringing, excitement for food, veterinary visits, and playing).

Two unrelated dogs (4.9%) had affected littermates with similar episodes.

### Ancillary diagnostic tests

Brain MRI was performed in 13/41 (31.7%), whole body CT in 1/41 (2.4%), and CSF analysis in 6/41 (14.6%); all results were unremarkable.

Abdominal ultrasonography was performed in 23/41 dogs (56.1%). Of these, 22 dogs had reported gastrointestinal signs, and 1 had dermatitis alone. Findings consistent with gastroenteropathy were observed in 21/23 (91.3%).

### AG and ATG2-A antibody titers

Antigliadin IgG titers were positive in 11/41 (26.8%), questionable in 7/41 (17.1%), and negative in 23/41 (56.1%).

Anti-transglutaminase-2 IgA titers were positive in 15/41 (36.6%), questionable in 6/41 (14.6%), and negative in 20/41 (48.8%).

When questionable results were considered negative, 21/41 dogs (51.2%) were seropositive for at least 1 antibody. Of these, 16/41 (39%) were positive for either AG or ATG2-A alone, whereas 5/41 (12.2%) were positive for both antibodies. The remaining 20/41 dogs (48.8%) were seronegative for both antibodies (see Table 1).

### Diet change and follow-up

All dogs, including those already receiving a GFD, were transitioned to a strict GFD consisting of either a commercial diet (27/41, 65.9%) or a home-prepared diet (14/41, 34.1%).

During short-term follow-up, most dogs responded to dietary modification, showing complete resolution in 24/41 (58.5%), and partial response in 15/41 (36.6%), regardless of antibody titers. In the remaining 2/41 dogs (4.9%), episode frequency was unchanged (1 yearly, 1 monthly), despite one being AG-positive. In both cases, no additional treatment was initiated, and no potential triggers were reported.

Among responders, 10/24 dogs (41.7%) with complete remission and 9/15 (60%) with partial response were seronegative for both antibodies (Table 2).

**Table 2:** Response to a GFD.

Variable	Complete	Partial	Unchanged
**Overall response (n = 41)**	24 (58.5%)	15 (36.6%)	2 (4.9%)
**Gastrointestinal signs (*n* = 33)**	22 (66.7%)	10 (30.3%)	1 (3%)
**No gastrointestinal signs (*n* = 8)**	2 (25%)	5 (62.5%)	1 (12.5%)
**Seropositive** ^**a**^ **(*n* = 21)**	14 (66.7%)	6 (28.6%)	1 (4.8%)
**Seronegative (*n* = 20)**	10 (50%)	9 (45%)	1 (5%)

Abbreviation: GFD = gluten-free diet.

a Seropositive: ≥ 1 positive antibody titer; questionable results were considered negative.

Gastrointestinal signs were reported in 22/24 dogs (91.7%) with complete remission and in 10/15 (66.7%) with partial response (Figure 1). All owners reported improvement of gastrointestinal signs after dietary change.

**Figure 1:**
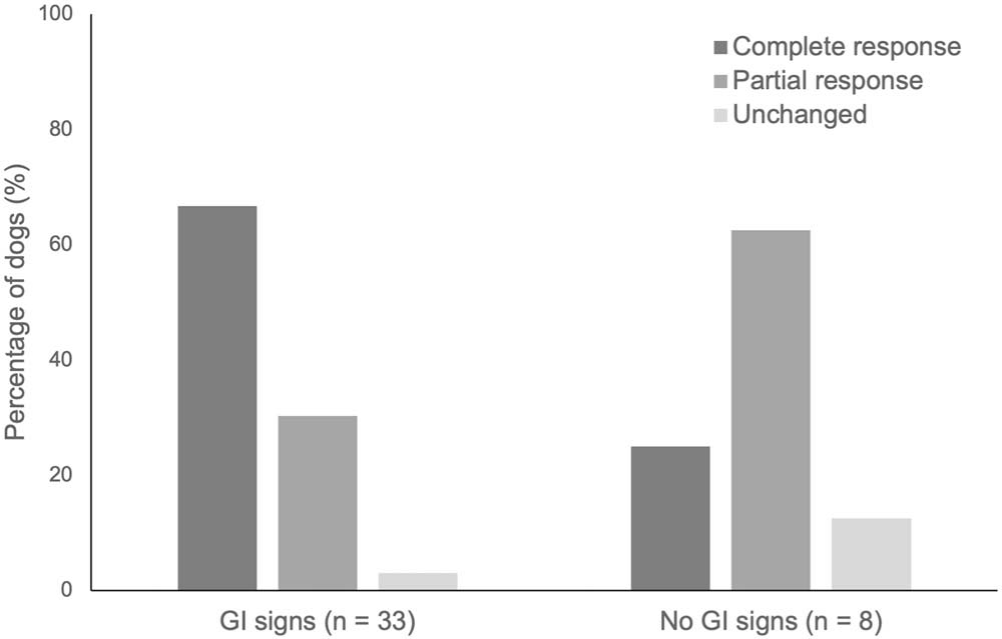
Response to a gluten-free diet in dogs with and without GI signs. Bars represent the percentage of dogs achieving complete, partial, or no response. Abbreviation: GI = gastrointestinal.

When evaluating response according to diet type, 19/27 dogs (70.4%) receiving a commercial GFD achieved complete resolution, 7/27 (25.9%) showed partial response, and 1/27 (3.7%) remained unchanged. In contrast, among dogs receiving a home-prepared diet, 5/14 (35.7%) achieved complete resolution, 8/14 (57.1%) showed partial response, and 1/14 (7.1%) remained unchanged (Table 3).

**Table 3:** Response to GFD according to diet type and antibody status.

Diet type	Complete	Partial	Unchanged
**Overall population (*n* = 41)**			
**Commercial GFD (*n* = 27)**	19 (70.4%)	7 (25.9%)	1 (3.7%)
**Home-prepared diet (*n* = 14)**	5 (35.7%)	8 (57.1%)	1 (7.1%)
**Seropositive** ^**a**^ **(*n* = 21)**			
**Commercial GFD (*n* = 15)**	12 (80%)	2 (13.3%)	1 (6.7%)
**Home-prepared diet (*n* = 6)**	2 (33.3%)	4 (66.7%)	–
**Seronegative (*n* = 20)**			
**Commercial GFD (*n* = 12)**	7 (58.3%)	5 (41.7%)	–
**Home-prepared diet (*n* = 8)**	3 (37.5%)	4 (50%)	1 (12.5%)

Abbreviation: GFD = gluten-free diet.

a Seropositive: ≥ 1 positive antibody titer; questionable results were considered negative.

When evaluating antibody status according to diet type, 15/27 dogs receiving a commercial GFD and 6/14 dogs receiving a home-prepared diet were seropositive. Among seropositive dogs, complete resolution was more frequent with commercial GFD (12/15, 80%) than with a home-prepared diet (2/6, 33%), whereas partial response was more frequent in dogs receiving a home-prepared diet (4/6, 66.7%) compared with those receiving a commercial diet (2/15, 13.3%). One seropositive dog receiving a commercial GFD (1/15, 6.7%) remained unchanged (Table 3).

In seronegative dogs, complete resolution also was more frequent with commercial GFD (7/12, 58.3%) than with a home-prepared diet (3/8, 37.5%), whereas partial response was slightly more frequent in the home-prepared diet group (4/8, 50%) compared with commercial diet (5/12, 41.7%). One seronegative dog receiving a home-prepared diet (1/8, 12.5%) remained unchanged (Table 3).

During short-term follow-up, 6 dogs had episodic relapses after being intentionally fed gluten-containing food by their owners.

During long-term follow-up, 9 dogs experienced relapses after an initial response to the GFD (complete resolution in 8, partial response in 1). Three dogs relapsed despite reported strict adherence to the GFD: relapse occurred after an initial complete remission lasting more than 8 months in 1 dog receiving a home-prepared diet and after a year in 1 dog receiving a commercial GFD. In the remaining dog, which was receiving a commercial GFD, relapse was associated with increased stress. Six dogs relapsed after deliberate administration of gluten-containing food by their owners: in 3, relapses were episodic; in 1, relapse was attributed to poor owner compliance; and in 2, initially episodic relapses became more frequent and were accompanied by worsening gastrointestinal signs.

Additional treatments were introduced in 6 dogs that, despite improvement after dietary change, continued to have frequent episodes (2 daily, 2 weekly, and 2 monthly). Antiseizure medications were prescribed in 5 dogs (phenobarbital in 2, levetiracetam in 3), with partial response in 2 (one dog with phenobarbital, improved from weekly to monthly; the other with levetiracetam, from daily to weekly). The remaining 3 dogs had ASM discontinued because of lack of efficacy. Fluoxetine was started in 1 dog, with complete remission during the first 2 months, followed by a return to the previous weekly frequency.

Among the 8 dogs with reported autonomic signs, 4 achieved complete resolution and 4 showed a partial response. In only 1 dog with partial response, levetiracetam was started, without improvement.

At ≥ 6 months follow-up, AG and ATG2-A antibody titers were re-evaluated in 5 dogs (3 with complete resolution, 2 of which relapsed after gluten ingestion, 1 with partial response, and 1 with unchanged episode frequency). All were negative for both antibodies. At first evaluation, 4/5 of these dogs had been positive for one or both antibodies, whereas 1/5 (the dog with unchanged frequency) was negative for both. Abdominal ultrasonography also was repeated in 2 of these dogs, both showing improvement of previously identified gastroenteropathy and complete resolution with GFD.

### Statistical associations

A summary of the statistical associations is provided in Table 4.

**Table 4:** Statistical associations.

Variables compared	Test	χ ^2^	df	*P*-value ^a^	Effect size (Cramer’s V)
**Breed × Episode frequency**	χ^2^ (Monte Carlo)	27.67	24	.27	0.47
**Breed × GFD response**	χ^2^ (Monte Carlo)	16.08	16	.42	0.44
**Antibody status** ^**b**^ **× Episode frequency**	χ^2^ (Monte Carlo)	2.40	3	.59	0.24
**Antibody status** ^**b**^ **× GI signs**	Fisher’s exact	–	–	1	0.05
**Antibody status** ^**b**^ **× GFD response**	χ^2^ (Monte Carlo)	1.24	2	.54	0.17
**GI signs × GFD response**	χ^2^ (Monte Carlo)	4.92	2	.08	0.35
**Antibody status** ^**c**^ **× Episode frequency**	χ^2^ (Monte Carlo)	2.59	3	.52	0.25
**Antibody status** ^**c**^ **× GI signs**	Fisher’s exact	–	–	1	0.05
**Antibody status** ^**c**^ **× GFD response**	χ^2^ (Monte Carlo)	3.36	1	.25	0.29
**Diet type × GFD response in overall population**	χ^2^ (Monte Carlo)	4.57	2	.09	0.33
**Diet type × GFD response in seropositive** ^**b**^ **dogs**	χ^2^ (Monte Carlo)	6.07	2	.03	0.54

Abbreviations: df = degree of freedom; GFD = gluten-free diet; GI = gastrointestinal.

a *P*-values < .05 were considered statistically significant. Monte Carlo p-values were used when expected cell counts were < 5.

b Questionable results were considered negative.

c Questionable results were considered positive.

No significant associations were found between breed and episode frequency (*P* = .27), or between breed and response to GFD (*P* = .42).

Antibody status was not significantly associated with episode frequency (*P* = .59), presence of gastrointestinal signs (*P* = 1), or response to GFD (*P* = .54). These results remained consistent when questionable antibody titers were alternatively classified as negative or positive.

No significant association was identified between the presence of gastrointestinal signs and response to a GFD (*P* = .08).

No significant association was identified between diet type (commercial versus home-prepared) and response to GFD in the overall population (*P* = .09). However, among seropositive dogs, diet type was significantly associated with response to GFD (*P* = .03). Dogs receiving commercial GFD showed a higher proportion of complete responses compared with those fed a home-prepared diet.

## Discussion

We demonstrated that most dogs with presumptive PD experienced clinical improvement after implementation of a GFD, regardless of antibody status. However, our hypothesis that response would be better in dogs with gastrointestinal signs or positive antibody titers was not statistically supported.

Paroxysmal dyskinesia has been increasingly recognized in dogs over the past decades, with Border Terriers being the first breed in which a gluten-sensitive form of PD was identified.^12–14^ Since then, beneficial effects of GFD have been observed and suspected in several other breeds, suggesting that gluten sensitivity may represent an underlying cause of PD in a broader population of dogs.^18^^,^^19^ Our study provides further evidence supporting a potential association between gluten sensitivity and PD across multiple breeds.

In humans, CD and NCGS are both gluten-related disorders that share intestinal and extra-intestinal manifestations and respond to dietary management, but differ in pathogenesis and diagnostic criteria. Celiac disease is an autoimmune enteropathy that occurs in genetically predisposed individuals and is diagnosed based on seropositivity for anti-tissue transglutaminase or anti-endomysium antibodies or both, characteristic histopathologic findings (the triad of villous atrophy, hyperplasia of the crypts, and intestinal intraepithelial lymphocytosis), and clinical response to a GFD. Nonceliac gluten sensitivity, on the other hand, is a less well-understood condition, with a suspected innate immune response, characterized by seropositivity for AG in some cases, absence of enteropathy, improvement with GFD, and relapse after gluten challenge.^20–26^

In dogs, PD appears to share similarities with both gluten-related disorders of humans. Histopathologic analysis of intestinal tissue would be useful to determine whether affected dogs display changes compatible with NCGS or CD.

In our study, several dogs with negative antibody titers still showed improvement after GFD implementation. It is possible that other, yet unidentified, antibodies or metabolites contribute to the pathogenesis of PD, as proposed in humans.^27^ Furthermore, there is ongoing debate regarding the distinction between NCGS and wheat sensitivity, because non-gluten components such as amylase-trypsin inhibitors or fermentable carbohydrates also may play a pathogenic role.^27^^,^^28^

In dogs, antibody seropositivity alone does not establish a diagnosis of PD, because it reflects an immune response to gliadin exposure but does not confirm that clinical signs are caused by gluten.^29^ Moreover, those dogs with equivocal antibody results are difficult to categorize as positive or negative, suggesting that breed-specific cut-off values might be needed. In human medicine, AG antibodies also can be detected in healthy individuals, which further limits their diagnostic specificity.^27^^,^^30^^,^^31^

Most dogs in our cohort exhibited concurrent gastrointestinal signs that improved after dietary modification. This finding supports a possible role of the GBA in PD pathogenesis and that the condition may involve multiple systems.^32^^,^^33^ The GBA represents a complex bidirectional communication network between the gastrointestinal tract and the central nervous system, mediated through neural, metabolic, immune, and endocrine pathways.^19^^,^^34^ Alterations in the GBA have been implicated in several neurologic conditions of humans, including epilepsy and gluten-related movement disorders, and similar mechanisms may be present in dogs.^17^^,^^19^^,^^35–40^ Whether the primary dysfunction originates in the gastrointestinal tract or the brain remains unclear, but our results support multisystem involvement, as also suggested by the presence of dermatopathies in some affected dogs.

The main differential diagnosis for PD in dogs is focal epileptic seizure, typically characterized by altered mentation, autonomic signs, and a postictal period. In our study, 8 dogs exhibited autonomic signs, but maintained awareness and responsiveness during episodes. Furthermore, all 8 responded to a GFD, with 4 achieving complete resolution. Although dietary response alone cannot rule out epileptic seizures, given the reported association between gluten-related disorders and epilepsy,^37^^,^^38^^,^^40^ the overall clinical features, long-term follow-up period, and the lack of ASM response in most cases were considered more supportive of PD. Additionally, although whole body or bilateral limb involvement observed in some dogs hypothetically could be compatible with generalized epileptic seizure activity, which would generally imply bilateral cortical involvement, the absence of altered mentation or a postictal phase made an epileptic origin less likely. In 5 dogs with salivation, concurrent gastrointestinal signs also were reported, suggesting a possible gastroenteric rather than epileptic origin, or a multisystem disorder as described elsewhere.^18^ Another dog showed transient apathy after episodes involving all 4 limbs, without autonomic signs, likely reflecting fatigue rather than a true postictal state. Nevertheless, a shared pathophysiologic pathway between epilepsy and PD cannot be entirely excluded, and EEG could help differentiate the 2 conditions in future studies.

A particularly challenging scenario is represented by Pomeranian dogs. A recent study^41^ showed that focal epileptic seizures in this breed may present with limb contraction, with EEG supporting an epileptic origin in many cases. In our cohort, all 7 Pomeranian dogs showed some degree of improvement after a GFD, with 2 achieving complete resolution with dietary change alone. Antiseizure medications were administered in 3 dogs, with only partial response in 2. Although epileptic seizures cannot be excluded in some dogs, particularly in the absence of EEG confirmation, these findings suggest that gluten-sensitive PD also should be considered in this breed. A pragmatic clinical approach could include an initial dietary trial, followed by ASM depending on episode severity and frequency.

Relapses were mainly associated with gluten re-exposure and often resolved once gluten-containing foods were eliminated. In a few cases, relapse was accompanied by concurrent worsening of gastrointestinal signs, further supporting potential involvement of the GBA. Three dogs relapsed despite strict adherence to a GFD; these relapses may have resulted from natural fluctuation of the episodes or inadvertent gluten intake. In one of these dogs, relapse appeared to be stress-associated, suggesting that behavioral management might be beneficial in selected cases, as reported in Scottish Terriers.^2^^,^^8^

During follow-up evaluation of antibody titers and abdominal ultrasonography, in all dogs with initially positive antibody titers, serology became negative at follow-up. Similarly, the 2 dogs that underwent repeat abdominal ultrasonography showed improvement of previously identified gastroenteropathy. These findings suggest that dietary modification may have led not only to neurologic improvement but also to normalization of gastrointestinal and serological abnormalities, reinforcing the suspected link between gluten sensitivity and PD, although only a few dogs were re-evaluated.

Pharmacological treatment for PD in dogs remains poorly defined. Among our cases, only mild improvement was observed in dogs treated with ASM or fluoxetine. Given the small number of pharmacologically treated dogs, no conclusions can be drawn regarding drug efficacy.

Dietary management appears to offer the most consistent clinical benefit, particularly in dogs with concurrent gastrointestinal signs. However, spontaneous remission of episodes cannot be excluded, as reported in other breeds.^9^ Gluten reintroduction would be necessary to confirm causality, but doing so carries a risk of relapse or aggravation of both neurologic and gastrointestinal signs, as observed in 2 dogs in our study.

Inadvertent gluten contamination of the diet, as reported in studies both in veterinary and human medicine,^42^^,^^43^ cannot be excluded as a contributor to the partial or absent response observed in some dogs. Although no significant association between diet type and response was identified in the overall population, a higher proportion of complete responses was observed in dogs receiving a commercial GFD compared with those receiving a home-prepared diet, raising the possibility of cross-contamination during food preparation. Moreover, among seropositive dogs, those receiving a commercial GFD showed a significantly higher proportion of complete responses than dogs receiving a home-prepared diet. These findings suggest that strict gluten avoidance may be more difficult to achieve with home-prepared diets, particularly in dogs with increased sensitivity to gluten exposure, as also described in human patients with gluten-related disorders.^43^^,^^44^ However, these results should be interpreted cautiously because of the small and unbalanced subgroup sizes.

Based on our results, a GFD may be considered as a first-line treatment in dogs with presumptive PD, regardless of antibody status. Although this approach may raise questions regarding the clinical utility of antibody testing, we currently recommend both antibody measurement and initiation of a GFD trial, including in seronegative dogs, given the limited evidence as to whether treatment should be guided by antibody status and the unclear utility of antibody testing.^14^^,^^18^^,^^19^

Our study had several limitations, including its retrospective design, lack of MRI evaluation in most dogs and EEG evaluation to differentiate PD from epileptic seizures, although the availability of video recordings and the long-term follow-up mitigated some of these limitations.

In conclusion, our study supports a potential association between gluten sensitivity and PD in dogs of various breeds, regardless of antibody titers. The favorable response to a GFD reinforces the possible role of the GBA in the disease pathogenesis. Commercial GFD may provide more consistent clinical responses than home-prepared diets, particularly in seropositive dogs. However, causality between gluten exposure and the paroxysmal episodes cannot be definitively established based on our study. Additional prospective, controlled studies including clinical scoring systems and serial serologic, electrophysiologic, and gastrointestinal assessments are warranted to better define diagnostic criteria and therapeutic strategies for PD in dogs.

## Supplementary Material

Supplementary_table_1_aalag238

## Data Availability

The data are not publicly available because of privacy and ethical restrictions.

## Off-label antimicrobial declaration

The authors declare no off-label use of antimicrobials.

## Institutional animal care and use committee or other approval declaration

Previous informed written consent was obtained. All procedures performed were done solely for the dog’s benefit and standard diagnostic and monitoring purposes and complied with the European legislation “on the protection of animals used for scientific purposes” (Directive 2010/63/EU) and with the ethical requirements of Italian law (Decreto Legislativo March 4, 2014, n. 26). Accordingly, this study did not require authorization or protocol number.

## Abbreviations

AG: anti-gliadin IgG
ASM: antiseizure medication
ATG2-A: anti-transglutaminase-2 immunoglobulin A
CD: celiac disease
CSF: cerebrospinal fluid
CT: computed tomography
EEG: electroencephalography
GBA: gut–brain axis
GFD: gluten-free diet
MRI: magnetic resonance imaging
NCGS: nonceliac gluten sensitivity
PD: paroxysmal dyskinesia
