# Supplementary material for: Presumptive paroxysmal dyskinesia in 41 dogs: is the diet change enough?
Source: J Vet Intern Med. 2026 Oct 9;40(5):aalag238. doi: 10.1093/jvimsj/aalag238 (PMC13652642; doi:10.1093/jvimsj/aalag238)
Supplement: Supplementary file 1 [file supplementary_table_1_aalag238.docx]

**Supplementary table 1. Detailed description of paroxysmal dyskinesia episodes in 41 dogs.** The table summarizes signalment, episode phenotype, duration, frequency, potential triggers, and presence of gastrointestinal signs for each dog.

| **Breed, sex & age at presentation** | **Episode phenotype** | **Episode duration** | **Episode frequency** | **Potential trigger?** | **GI signs?** |
| --- | --- | --- | --- | --- | --- |
| Crossbreed, MN, 7.5 yo | Dystonia in the thoracic limbs, head tremors, kyphosis, frequent falls when trying to walk. The dog appeared anxious during episodes. | 1-2 min | weekly | no | yes |
| Crossbreed, MN, 7 yo | Dystonia in the thoracic limbs, head and whole-body tremors, mostly in lateral recumbency, difficulty walking. | 1-60 min | daily | yes | yes |
| Crossbreed, MI, 5 yo | Dystonia in all four limbs, unsuccessful attempts to walk. | 3 min | monthly | no | yes |
| Crossbreed, MN, 4.6 yo | Dystonia in all four limbs, predominantly in the pelvic limbs, unsuccessful attempts to walk. | 5 min | monthly | no | yes |
| Crossbreed, MI, 7.1 yo | Rigidity of all four limbs, in lateral recumbency or standing, vestibulo-cerebellar ataxia, continuous licking, difficulty walking, once urination of a small amount. The dog appeared anxious during episodes. | 2-5 min | yearly | no | yes |
| Crossbreed, MN, 10.8 yo | Dystonia in the limbs on one side, in lateral recumbency, difficulty walking. The dog appeared anxious and painful during episodes. | 1-2 min | monthly | no | no |
| Crossbreed, FI, 4.5 yo | Dystonia in the pelvic limbs, kyphosis, stiff tail, lying down, difficulty walking. The dog appeared anxious and painful during episodes. | 3-4 min | monthly | no | yes |
| Crossbreed, MI, 7.3 yo | Whole-body tremors, truncal swaying, difficulty walking. The dog appeared anxious during episodes. | 2-4 min | daily | yes | yes |
| Crossbreed, FN, 8.8 yo | Dystonia of one thoracic limb, continuous licking, difficulty walking. The dog appeared anxious and painful during episodes. | 1-2 min | monthly | no | yes |
| Poodle, MI, 5.5 yo | Stiffness of body and all four limbs, truncal dystonia, whole-body tremor, difficulty walking. | 2 min | monthly | no | yes |
| Poodle, FN, 6 yo | Dystonia in all four limbs, predominantly in the pelvic limbs, crouching gait, salivation. | 1-2 min | monthly | no | yes |
| Poodle, MI, 4.2 yo | Dystonia in all four limbs, predominantly in the pelvic limbs, kyphosis, difficulty walking. The dog appeared anxious and painful during episodes. | 15 min | monthly | no | yes |
| Poodle, MI, 5.8 yo | Dystonia in all four limbs, kyphosis, truncal swaying, difficulty walking. The dog appeared anxious during episodes. | 5 min | weekly | no | yes |
| Poodle, FS, 4.3 yo | Dystonia of one limb (alternating between a thoracic or, mainly, pelvic limb), truncal swaying, continuous licking, unsuccessful attempts to walk. The dog sometimes appeared painful during episodes. | 2-3 min | daily | no | yes |
| Poodle, FI, 3 yo | Dystonia in all four limbs, predominantly in the thoracic limbs, severe head tremors, sometimes whole-body tremors, difficulty walking. The dog appeared anxious during episodes. | 10-15 min | monthly | no | yes |
| Poodle, MN, 7 yo | Dystonia in all four limbs, predominantly in the thoracic limbs, severe head tremors, sometimes kyphosis, frequent falls when trying to walk. The dog appeared anxious during episodes. | 10-60 min | yearly | no | yes |
| Maltese, FI, 7 yo | Rigidity of all four limbs, difficulty walking. The dog appeared painful during episodes. | 2-6 hours | yearly | no | yes |
| Maltese, MN, 8.5 yo | Dystonia in all four limbs, wide head movements, unsuccessful attempts to walk, salivation and urination. The dog appeared anxious during episodes. | 2-10 min | monthly | no | yes |
| Maltese, FI, 4.4 yo | Dystonia in all four limbs, predominantly in the pelvic limbs, difficulty walking. The dog appeared anxious and painful during episodes. | 2-3 min | daily | no | yes |
| Maltese, FN, 5 yo | Dystonia in all four limbs, sometimes only in the thoracic limbs, kyphosis, truncal swaying, whole-body tremors, difficulty walking. The dog appeared anxious during episodes. | 3-5 min | monthly | no | yes |
| Maltese, MI, 9 yo | Dystonia in the pelvic limbs, whole-body tremors, truncal swaying, lowered head carriage with a stiff neck, difficulty walking. The dog appeared anxious during episodes. | 2-3 min | daily | no | no |
| Maltese, MN, 7.1 yo | Dystonia of one thoracic limb, difficulty walking. | 3-4 min | daily | no | no |
| Maltese, FN, 5 yo | Dystonia in all four limbs, kyphosis, truncal swaying, difficulty walking. | < 1 min | monthly | no | yes |
| Pomeranian, MI, 7 yo | Cramps in all four limbs, difficulty walking. | 3 min | weekly | no | yes |
| Pomeranian, FI, 0.5 yo | Dystonia in all four limbs (sometimes only one limb), whole-body tremors, vestibulo-cerebellar ataxia, difficulty walking, sometimes salivation. The dog appeared anxious and painful during episodes. | 3-60 min | daily | no | no |
| Pomeranian, MI, 7.5 yo | Dystonia in all four limbs (mainly on one side), in sternal recumbency, difficulty walking, salivation, sometimes urination and defecation. | 1-2 min | daily | no | yes |
| Pomeranian, MI, 8.5 yo | Dystonia in all four limbs, whole-body tremors, difficulty walking. The dog appeared anxious and painful during episodes. | 2 min | daily | yes | yes |
| Pomeranian, MI, 0.8 yo | Dystonia of one limb (alternating between a thoracic or pelvic limb), in sternal recumbency, whole-body and head (mainly) tremors, difficulty walking. The dog appeared anxious and painful during episodes. | 1-2 min | monthly | no | no |
| Pomeranian, FN, 4.4 yo | Dystonia in the pelvic limbs, in sternal recumbency, unsuccessful attempts to stand up and to walk. The dog appeared anxious and painful during episodes. | 1-2 min | monthly | no | yes |
| Pomeranian, MI, 6.5 yo | Dystonia of one limb (alternating a thoracic or pelvic limb) while walking, sometimes eyes twitching, difficulty walking. The dog appeared anxious during episodes. | 5-60 min | daily | no | yes |
| Labrador Retriever, MN, 6.7 yo | Dystonia in the pelvic limbs while standing, difficulty walking. | 2-5 min | monthly | yes | no |
| Labrador Retriever, MI, 4.5 yo | Dystonia in all four limbs, in lateral recumbency, difficulty walking. The dog appeared anxious during episodes. Mild apathy after episodes. | < 1 min | monthly | no | yes |
| Labrador Retriever, MI, 5 yo | Dystonia in all four limbs, in lateral recumbency, whole-body and head tremors, difficulty walking. The dog appeared anxious and painful during episodes. | 2-40 min | yearly | yes | yes |
| Labrador Retriever, MI, 7.3 yo | Dystonia of one thoracic limb, in lateral recumbency, whole-body tremors, difficulty walking, mild salivation. The dog appeared anxious during episodes. | 2 min | yearly | no | yes |
| French Bulldog, MI, 2 yo | Vestibulo-cerebellar ataxia, whole-body tremors, difficulty walking, sometimes salivation. | 4 min | monthly | no | no |
| French Bulldog, FN, 2.5 yo | Cramps in all four limbs, difficulty walking. | 3-4 min | monthly | yes | no |
| French Bulldog, FN, 3.3 yo | Rigidity of the pelvic limbs, vestibulo-cerebellar ataxia, difficulty walking, mild salivation and urination. The dog appeared anxious during episodes. | < 1 min | yearly | no | yes |
| Jack Russell Terrier, FN, 4.6 yo | Stiffness of body and all four limbs, difficulty walking. The dog appeared anxious during episodes. | 120-360 min | yearly | no | yes |
| Jack Russell Terrier, FN, 2.8 yo | Dystonia in all four limbs, in sternal recumbency, difficulty walking. | 1-2 min | weekly | yes | yes |
| Chihuahua, MI, 6 yo | Dystonia in the pelvic limbs, kyphosis, difficulty walking. | 5 min | weekly | no | yes |
| Cavalier King Charles Spaniel, MI, 7 yo | Rigidity of all four limbs, in lateral recumbency, difficulty walking. The dog appeared anxious during episodes. | 30 min | yearly | no | yes |

FI: female intact; FN: female neutered; MI: male intact; MN: male neutered; GI: gastrointestinal; yo: years-old.
